# *N* ^4^-(3-Bromo­phen­yl)quinazoline-4,6-diamine

**DOI:** 10.1107/S1600536810002631

**Published:** 2010-01-27

**Authors:** De-Liang Li, Yang Wu, Qiang Wang, Gu He, Luo-Ting Yu

**Affiliations:** a State Key Laboratory of Biotherapy and Cancer Center, West China Hospital, West China Medical School, Sichuan University, Chengdu 610041, People’s Republic of China

## Abstract

In the title compound, C_14_H_11_BrN_4_, the fused benzene and pyrimidine rings are nearly coplanar, making dihedral angles of 1.26 (14) and 3.53 (15)° in the two independent mol­ecules. In the crystal structure, π–π stacking inter­actions [centroid–centroid distances = 3.4736 (19) and 3.5416 (19) Å] and weak N—H⋯N and N—H⋯Br inter­actions contribute to the stability of the structure.

## Related literature

For general background to the biological activity of *N* ^4^-(3-bromo­phen­yl)quinazoline derivatives, see: Fry *et al.* (2005 ▶).
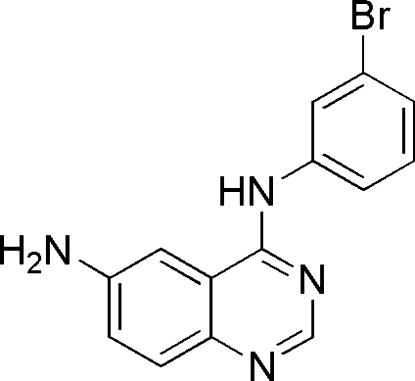


## Experimental

### 

#### Crystal data

C_14_H_11_BrN_4_
*M* *_r_* = 315.18
Triclinic, 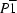

*a* = 7.5579 (15) Å
*b* = 11.743 (2) Å
*c* = 15.554 (3) Å
α = 110.24 (3)°
β = 96.79 (3)°
γ = 96.75 (3)°
*V* = 1267.4 (4) Å^3^
*Z* = 4
Mo *K*α radiation
μ = 3.23 mm^−1^
*T* = 113 K
0.36 × 0.26 × 0.23 mm

#### Data collection

Rigaku Saturn CCD area-detector diffractometer
Absorption correction: multi-scan [*SADABS* (Sheldrick, 1996 ▶) using a modified Dwiggins (1975 ▶) procedure] *T*_min_ = 0.389, *T*_max_ = 0.523
10616 measured reflections
5931 independent reflections
3735 reflections with *I* > 2σ(*I*)
*R*_int_ = 0.034

#### Refinement

*R*[*F* ^2^ > 2σ(*F* ^2^)] = 0.043
*wR*(*F* ^2^) = 0.110
*S* = 1.02
5931 reflections
360 parameters
H atoms treated by a mixture of independent and constrained refinement
Δρ_max_ = 1.42 e Å^−3^
Δρ_min_ = −1.54 e Å^−3^

### 

Data collection: *CrystalClear* (Rigaku/MSC, 2005 ▶); cell refinement: *CrystalClear*; data reduction: *CrystalClear*; program(s) used to solve structure: *SHELXS97* (Sheldrick, 2008 ▶); program(s) used to refine structure: *SHELXL97* (Sheldrick, 2008 ▶); molecular graphics: *ORTEPIII* (Burnett & Johnson, 1996 ▶); software used to prepare material for publication: *PLATON* (Spek, 2009 ▶).

## Supplementary Material

Crystal structure: contains datablocks global, I. DOI: 10.1107/S1600536810002631/pb2020sup1.cif

Structure factors: contains datablocks I. DOI: 10.1107/S1600536810002631/pb2020Isup2.hkl

Additional supplementary materials:  crystallographic information; 3D view; checkCIF report

## Figures and Tables

**Table 1:** Hydrogen-bond geometry (Å, °)

*D*—H⋯*A*	*D*—H	H⋯*A*	*D*⋯*A*	*D*—H⋯*A*
N1—H1*N*⋯N4^i^	0.93 (4)	2.28 (4)	3.069 (4)	142 (3)
N4—H4*A*⋯N3^ii^	0.88	2.33	3.137 (4)	153
N4—H4*B*⋯N8^iii^	0.88	2.39	3.178 (4)	149
N8—H8*N*1⋯Br1^iv^	0.89 (4)	2.88 (4)	3.739 (4)	163 (3)
N8—H8*N*2⋯N7^ii^	0.77 (4)	2.31 (4)	3.053 (4)	162 (4)

Symmetry codes: (i) 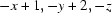; (ii) 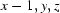; (iii) 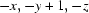; (iv) 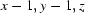.

**Fig. 1.**
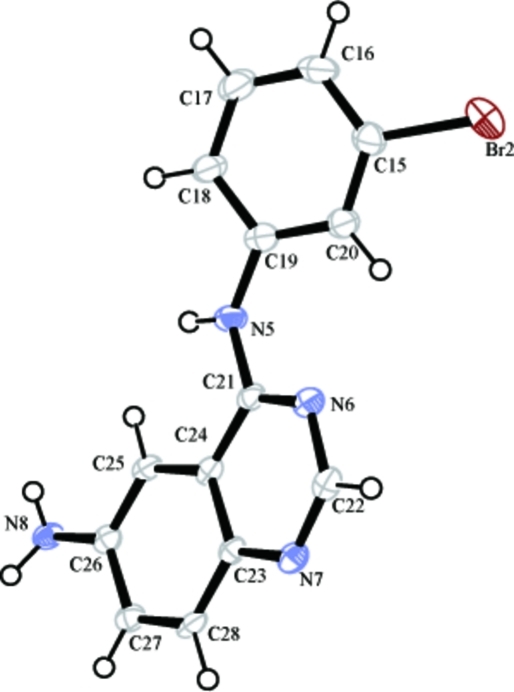
The molecular structure of the title compound, with displacement ellipsoids drawn at the 30% probability level.

**Fig. 2.**
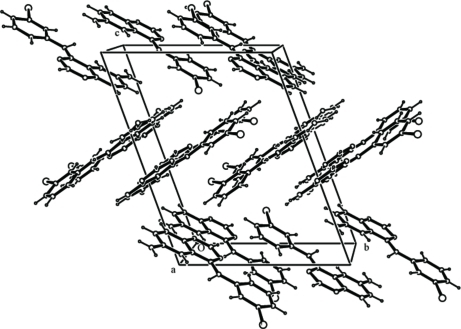
A packing diagram of the title compound.

## supplementary crystallographic information

### Comment

N4-(3-bromophenyl)quinazoline derivatives are of great importance owing to their wide biological properties (Fry *et al.* 1994). The title compound is one of the key intermediates in our synthetic investigations of antitumor drugs. We report here its crystal structure. As shown in Fig. 1, the benzene and pyrimidine rings of the title compound (I) are nearly coplanar, with the dihedral angle between them are 1.2° and 3.1°, respectively. A combination of intermolecular π-π packing interaction, N—H···N and N—H···Br hydrogen bonds plays important part in the connection of adjacent molecules.

### Experimental

A mixture of N4-(3-bromophenyl)quinazoline-4,6-diamine (3.45 g, 10 mmol), Sodium sulfide nonahydrate (6.00 g, 25 mmol), sodium hydroxide (2.00 g, 50 mmol), ethanol (40 ml) and water (80 ml) was heated for 5.0 h under reflux. The ethanol was removed under vacuum. The solid was filtered, washed with cold water, dried to yield the title compound as a brown solid (2.2 g, 71% yield). Crystals suitable for X-ray analysis were obtained by slow evaporation from a solution of ethyl acetate.

### Refinement

H atoms of the amino group were located in a difference map and refined freely. The reminaing H atoms were positioned geometrically (C—H = 0.93–0.97 Å) and refined using a riding model, with *U*_iso_(H) = 1.2–1.5*U*_eq_(C).

### Crystal data

**Table:**

C_14_H_11_BrN_4_	*Z* = 4
*M**_r_* = 315.18	*F*(000) = 632
Triclinic, *P*1	*D*_x_ = 1.652 Mg m^−^^3^
Hall symbol: -P 1	Mo *K*α radiation, λ = 0.71073 Å
*a* = 7.5579 (15) Å	Cell parameters from 3726 reflections
*b* = 11.743 (2) Å	θ = 1.9–27.9°
*c* = 15.554 (3) Å	µ = 3.23 mm^−^^1^
α = 110.24 (3)°	*T* = 113 K
β = 96.79 (3)°	Block, colourless
γ = 96.75 (3)°	0.36 × 0.26 × 0.23 mm
*V* = 1267.4 (4) Å^3^	

### Data collection

**Table:**

Rigaku Saturn CCD area-detector diffractometer	5931 independent reflections
Radiation source: rotating anode	3735 reflections with *I* > 2σ(*I*)
confocal	*R*_int_ = 0.034
Detector resolution: 7.31 pixels mm^-1^	θ_max_ = 27.9°, θ_min_ = 1.9°
ω and φ scans	*h* = −9→9
Absorption correction: multi-scan [*SADABS* (Sheldrick, 1996) using a modified Dwiggins (1975) procedure]	*k* = −9→15
*T*_min_ = 0.389, *T*_max_ = 0.523	*l* = −20→20
10616 measured reflections	

### Refinement

**Table:**

Refinement on *F*^2^	Secondary atom site location: difference Fourier map
Least-squares matrix: full	Hydrogen site location: mixed
*R*[*F*^2^ > 2σ(*F*^2^)] = 0.043	H atoms treated by a mixture of independent and constrained refinement
*wR*(*F*^2^) = 0.110	*w* = 1/[σ^2^(*F*_o_^2^) + (0.052*P*)^2^] where *P* = (*F*_o_^2^ + 2*F*_c_^2^)/3
*S* = 1.02	(Δ/σ)_max_ = 0.001
5931 reflections	Δρ_max_ = 1.42 e Å^−^^3^
360 parameters	Δρ_min_ = −1.54 e Å^−^^3^
0 restraints	Extinction correction: *SHELXL97* (Sheldrick, 2008), Fc^*^=kFc[1+0.001xFc^2^λ^3^/sin(2θ)]^-1/4^
Primary atom site location: structure-invariant direct methods	Extinction coefficient: 0.0265 (14)

### Special details

**Table:**

Experimental. Absorption correction: [interpolation using *International Tables for Crystallography* (Vol. C, 1992, p. 523, Table 6.3.3.3) for values of µ*R* in the range 0–2.5, and *International Tables for X-ray Crystallography* (Vol. II, 1959, p. 302, Table 5.3.6B) for µ*R* in the range 2.6–10.0; the interpolation procedure of Dwiggins (1975) was used with some modification]
Geometry. All e.s.d.'s (except the e.s.d. in the dihedral angle between two l.s. planes) are estimated using the full covariance matrix. The cell e.s.d.'s are taken into account individually in the estimation of e.s.d.'s in distances, angles and torsion angles; correlations between e.s.d.'s in cell parameters are only used when they are defined by crystal symmetry. An approximate (isotropic) treatment of cell e.s.d.'s is used for estimating e.s.d.'s involving l.s. planes.
Refinement. Refinement of *F*^2^ against ALL reflections. The weighted *R*-factor *wR* and goodness of fit *S* are based on *F*^2^, conventional *R*-factors *R* are based on *F*, with *F* set to zero for negative *F*^2^. The threshold expression of *F*^2^ > σ(*F*^2^) is used only for calculating *R*-factors(gt) *etc*. and is not relevant to the choice of reflections for refinement. *R*-factors based on *F*^2^ are statistically about twice as large as those based on *F*, and *R*- factors based on ALL data will be even larger.

### Fractional atomic coordinates and isotropic or equivalent isotropic displacement parameters (Å^2^)

**Table:**

	*x*	*y*	*z*	*U*_iso_*/*U*_eq_	
Br1	0.32081 (4)	0.54984 (3)	0.19450 (3)	0.02528 (13)	
N1	0.7074 (3)	0.7482 (2)	0.01210 (19)	0.0175 (6)	
H1N	0.622 (5)	0.800 (4)	0.026 (3)	0.036 (11)*	
N2	0.9911 (3)	0.7443 (2)	−0.03199 (19)	0.0176 (6)	
N3	1.0997 (3)	0.8702 (2)	−0.11423 (19)	0.0175 (6)	
N4	0.4365 (3)	1.0316 (2)	−0.13941 (19)	0.0184 (6)	
H4A	0.3481	1.0082	−0.1139	0.022*	
H4B	0.4225	1.0832	−0.1684	0.022*	
C1	0.5318 (4)	0.5673 (3)	0.1418 (2)	0.0179 (7)	
C2	0.6659 (4)	0.4991 (3)	0.1522 (2)	0.0204 (7)	
H2	0.6528	0.4433	0.1839	0.024*	
C3	0.8198 (4)	0.5164 (3)	0.1143 (2)	0.0198 (7)	
H3	0.9136	0.4711	0.1204	0.024*	
C4	0.8412 (4)	0.5974 (3)	0.0679 (2)	0.0180 (7)	
H4	0.9484	0.6074	0.0431	0.022*	
C5	0.7044 (4)	0.6642 (3)	0.0580 (2)	0.0164 (7)	
C6	0.5474 (4)	0.6477 (3)	0.0953 (2)	0.0167 (7)	
H6	0.4524	0.6918	0.0885	0.020*	
C7	0.8344 (4)	0.7850 (3)	−0.0327 (2)	0.0158 (7)	
C8	1.1141 (4)	0.7914 (3)	−0.0728 (2)	0.0182 (7)	
H8	1.2267	0.7632	−0.0710	0.022*	
C9	0.9338 (4)	0.9078 (3)	−0.1195 (2)	0.0151 (6)	
C10	0.7933 (4)	0.8659 (3)	−0.0804 (2)	0.0149 (6)	
C11	0.6255 (4)	0.9061 (3)	−0.0889 (2)	0.0166 (7)	
H11	0.5295	0.8771	−0.0633	0.020*	
C12	0.5995 (4)	0.9869 (3)	−0.1338 (2)	0.0154 (7)	
C13	0.7425 (4)	1.0290 (3)	−0.1721 (2)	0.0196 (7)	
H13	0.7256	1.0854	−0.2026	0.024*	
C14	0.9041 (4)	0.9895 (3)	−0.1658 (2)	0.0191 (7)	
H14	0.9980	1.0175	−0.1929	0.023*	
Br2	0.47743 (6)	0.69252 (4)	0.64544 (4)	0.05308 (17)	
N5	0.0018 (4)	0.2805 (3)	0.5104 (2)	0.0236 (7)	
H5N	−0.105 (6)	0.250 (4)	0.497 (3)	0.062 (16)*	
N6	0.2839 (4)	0.2212 (3)	0.5094 (2)	0.0225 (6)	
N7	0.3236 (3)	0.0159 (3)	0.4224 (2)	0.0220 (6)	
N8	−0.4215 (4)	−0.1290 (3)	0.3066 (2)	0.0208 (6)	
H8N1	−0.470 (5)	−0.207 (4)	0.292 (3)	0.031 (11)*	
H8N2	−0.467 (5)	−0.080 (4)	0.339 (3)	0.029 (12)*	
C15	0.2510 (5)	0.5963 (3)	0.6368 (3)	0.0297 (8)	
C16	0.1242 (5)	0.6528 (4)	0.6842 (3)	0.0345 (9)	
H16	0.1503	0.7376	0.7228	0.041*	
C17	−0.0417 (5)	0.5833 (4)	0.6743 (3)	0.0353 (9)	
H17	−0.1317	0.6202	0.7067	0.042*	
C18	−0.0788 (5)	0.4601 (3)	0.6177 (3)	0.0294 (8)	
H18	−0.1943	0.4132	0.6112	0.035*	
C19	0.0523 (5)	0.4039 (3)	0.5700 (2)	0.0237 (8)	
C20	0.2198 (5)	0.4734 (3)	0.5799 (3)	0.0270 (8)	
H20	0.3112	0.4375	0.5483	0.032*	
C21	0.1078 (4)	0.1919 (3)	0.4798 (2)	0.0200 (7)	
C22	0.3821 (4)	0.1307 (3)	0.4780 (2)	0.0237 (8)	
H22	0.5091	0.1530	0.4988	0.028*	
C23	0.1403 (4)	−0.0162 (3)	0.3923 (2)	0.0176 (7)	
C24	0.0232 (4)	0.0702 (3)	0.4192 (2)	0.0179 (7)	
C25	−0.1655 (4)	0.0310 (3)	0.3887 (2)	0.0209 (7)	
H25	−0.2446	0.0894	0.4047	0.025*	
C26	−0.2359 (4)	−0.0901 (3)	0.3363 (2)	0.0200 (7)	
C27	−0.1169 (4)	−0.1752 (3)	0.3086 (2)	0.0208 (7)	
H27	−0.1645	−0.2585	0.2707	0.025*	
C28	0.0670 (4)	−0.1386 (3)	0.3358 (2)	0.0209 (7)	
H28	0.1452	−0.1968	0.3162	0.025*	

### Atomic displacement parameters (Å^2^)

**Table:**

	*U*^11^	*U*^22^	*U*^33^	*U*^12^	*U*^13^	*U*^23^
Br1	0.0237 (2)	0.0263 (2)	0.0295 (2)	0.00205 (15)	0.01249 (15)	0.01294 (17)
N1	0.0181 (13)	0.0173 (14)	0.0246 (17)	0.0096 (12)	0.0100 (12)	0.0127 (13)
N2	0.0158 (13)	0.0171 (14)	0.0222 (16)	0.0055 (11)	0.0069 (11)	0.0078 (13)
N3	0.0127 (12)	0.0170 (14)	0.0236 (16)	0.0032 (11)	0.0076 (11)	0.0068 (13)
N4	0.0159 (13)	0.0229 (15)	0.0244 (17)	0.0091 (12)	0.0096 (11)	0.0148 (14)
C1	0.0198 (16)	0.0165 (16)	0.0178 (18)	0.0030 (13)	0.0068 (13)	0.0058 (15)
C2	0.0245 (17)	0.0170 (17)	0.023 (2)	0.0053 (14)	0.0063 (14)	0.0095 (16)
C3	0.0224 (17)	0.0168 (17)	0.0229 (19)	0.0085 (14)	0.0046 (14)	0.0085 (15)
C4	0.0146 (15)	0.0190 (17)	0.0223 (19)	0.0058 (13)	0.0055 (13)	0.0082 (16)
C5	0.0207 (16)	0.0130 (15)	0.0144 (18)	0.0011 (13)	0.0053 (13)	0.0037 (14)
C6	0.0181 (16)	0.0149 (16)	0.0170 (18)	0.0061 (13)	0.0058 (13)	0.0036 (14)
C7	0.0145 (15)	0.0128 (15)	0.0182 (18)	0.0010 (13)	0.0063 (13)	0.0025 (14)
C8	0.0141 (15)	0.0147 (16)	0.027 (2)	0.0059 (13)	0.0073 (13)	0.0059 (15)
C9	0.0158 (15)	0.0131 (15)	0.0158 (17)	0.0030 (13)	0.0051 (12)	0.0036 (14)
C10	0.0150 (15)	0.0130 (15)	0.0163 (18)	0.0034 (13)	0.0058 (12)	0.0034 (14)
C11	0.0165 (15)	0.0152 (16)	0.0203 (19)	0.0038 (13)	0.0078 (13)	0.0075 (15)
C12	0.0137 (15)	0.0149 (16)	0.0163 (18)	0.0029 (13)	0.0025 (12)	0.0041 (14)
C13	0.0217 (17)	0.0207 (17)	0.0217 (19)	0.0057 (14)	0.0085 (14)	0.0120 (16)
C14	0.0164 (16)	0.0224 (18)	0.0209 (19)	0.0026 (14)	0.0082 (13)	0.0095 (16)
Br2	0.0563 (3)	0.0275 (2)	0.0698 (4)	−0.0053 (2)	0.0215 (3)	0.0114 (2)
N5	0.0242 (16)	0.0195 (16)	0.0269 (18)	0.0083 (14)	0.0065 (13)	0.0060 (14)
N6	0.0227 (15)	0.0241 (16)	0.0220 (17)	0.0070 (13)	0.0061 (12)	0.0084 (14)
N7	0.0180 (14)	0.0264 (16)	0.0234 (17)	0.0076 (13)	0.0052 (12)	0.0095 (14)
N8	0.0166 (14)	0.0225 (17)	0.0203 (18)	0.0056 (14)	0.0022 (12)	0.0037 (15)
C15	0.040 (2)	0.025 (2)	0.025 (2)	0.0030 (17)	0.0080 (17)	0.0102 (18)
C16	0.061 (3)	0.0206 (19)	0.024 (2)	0.0124 (19)	0.0130 (19)	0.0071 (18)
C17	0.047 (2)	0.035 (2)	0.030 (2)	0.015 (2)	0.0186 (19)	0.011 (2)
C18	0.038 (2)	0.026 (2)	0.028 (2)	0.0114 (17)	0.0147 (17)	0.0081 (18)
C19	0.0319 (19)	0.0238 (19)	0.019 (2)	0.0098 (16)	0.0056 (15)	0.0098 (17)
C20	0.036 (2)	0.0237 (19)	0.026 (2)	0.0098 (17)	0.0119 (16)	0.0119 (18)
C21	0.0244 (17)	0.0224 (18)	0.0176 (19)	0.0081 (15)	0.0091 (14)	0.0096 (16)
C22	0.0206 (17)	0.030 (2)	0.025 (2)	0.0069 (15)	0.0076 (14)	0.0129 (18)
C23	0.0187 (16)	0.0224 (18)	0.0144 (18)	0.0057 (14)	0.0046 (13)	0.0086 (15)
C24	0.0177 (16)	0.0239 (18)	0.0180 (18)	0.0092 (14)	0.0056 (13)	0.0121 (16)
C25	0.0221 (17)	0.0228 (18)	0.0201 (19)	0.0094 (15)	0.0079 (14)	0.0074 (16)
C26	0.0229 (17)	0.0239 (18)	0.0162 (19)	0.0067 (15)	0.0046 (13)	0.0094 (16)
C27	0.0242 (17)	0.0228 (18)	0.0160 (18)	0.0065 (15)	0.0050 (13)	0.0067 (15)
C28	0.0245 (17)	0.0270 (19)	0.0199 (19)	0.0160 (15)	0.0120 (14)	0.0128 (16)

### Geometric parameters (Å, °)

**Table:**

Br1—C1	1.899 (3)	Br2—C15	1.900 (4)
N1—C7	1.365 (4)	N5—C21	1.376 (4)
N1—C5	1.403 (4)	N5—C19	1.401 (5)
N1—H1N	0.93 (4)	N5—H5N	0.82 (4)
N2—C7	1.328 (4)	N6—C21	1.319 (4)
N2—C8	1.354 (4)	N6—C22	1.353 (4)
N3—C8	1.305 (4)	N7—C22	1.313 (4)
N3—C9	1.380 (4)	N7—C23	1.372 (4)
N4—C12	1.400 (4)	N8—C26	1.392 (4)
N4—H4A	0.8800	N8—H8N1	0.89 (4)
N4—H4B	0.8800	N8—H8N2	0.77 (4)
C1—C6	1.377 (4)	C15—C16	1.372 (5)
C1—C2	1.391 (4)	C15—C20	1.381 (5)
C2—C3	1.390 (4)	C16—C17	1.374 (5)
C2—H2	0.9500	C16—H16	0.9500
C3—C4	1.384 (4)	C17—C18	1.383 (5)
C3—H3	0.9500	C17—H17	0.9500
C4—C5	1.395 (4)	C18—C19	1.399 (5)
C4—H4	0.9500	C18—H18	0.9500
C5—C6	1.403 (4)	C19—C20	1.384 (5)
C6—H6	0.9500	C20—H20	0.9500
C7—C10	1.433 (4)	C21—C24	1.431 (5)
C8—H8	0.9500	C22—H22	0.9500
C9—C10	1.406 (4)	C23—C28	1.402 (5)
C9—C14	1.407 (4)	C23—C24	1.412 (4)
C10—C11	1.412 (4)	C24—C25	1.416 (4)
C11—C12	1.378 (4)	C25—C26	1.374 (5)
C11—H11	0.9500	C25—H25	0.9500
C12—C13	1.414 (4)	C26—C27	1.413 (4)
C13—C14	1.362 (4)	C27—C28	1.375 (4)
C13—H13	0.9500	C27—H27	0.9500
C14—H14	0.9500	C28—H28	0.9500
			
C7—N1—C5	131.2 (2)	C21—N5—C19	129.5 (3)
C7—N1—H1N	114 (2)	C21—N5—H5N	111 (3)
C5—N1—H1N	113 (2)	C19—N5—H5N	119 (3)
C7—N2—C8	115.9 (2)	C21—N6—C22	116.5 (3)
C8—N3—C9	115.2 (3)	C22—N7—C23	116.0 (3)
C12—N4—H4A	120.0	C26—N8—H8N1	121 (2)
C12—N4—H4B	120.0	C26—N8—H8N2	106 (3)
H4A—N4—H4B	120.0	H8N1—N8—H8N2	117 (4)
C6—C1—C2	122.4 (3)	C16—C15—C20	123.2 (4)
C6—C1—Br1	118.8 (2)	C16—C15—Br2	118.7 (3)
C2—C1—Br1	118.8 (2)	C20—C15—Br2	118.1 (3)
C1—C2—C3	117.0 (3)	C15—C16—C17	118.1 (4)
C1—C2—H2	121.5	C15—C16—H16	121.0
C3—C2—H2	121.5	C17—C16—H16	121.0
C4—C3—C2	122.3 (3)	C16—C17—C18	120.6 (4)
C4—C3—H3	118.9	C16—C17—H17	119.7
C2—C3—H3	118.9	C18—C17—H17	119.7
C3—C4—C5	119.5 (3)	C17—C18—C19	120.5 (4)
C3—C4—H4	120.2	C17—C18—H18	119.7
C5—C4—H4	120.2	C19—C18—H18	119.7
C4—C5—C6	119.1 (3)	C20—C19—C18	119.1 (3)
C4—C5—N1	125.6 (3)	C20—C19—N5	123.6 (3)
C6—C5—N1	115.3 (3)	C18—C19—N5	117.2 (3)
C1—C6—C5	119.6 (3)	C15—C20—C19	118.5 (3)
C1—C6—H6	120.2	C15—C20—H20	120.7
C5—C6—H6	120.2	C19—C20—H20	120.7
N2—C7—N1	119.6 (3)	N6—C21—N5	118.7 (3)
N2—C7—C10	121.8 (3)	N6—C21—C24	122.1 (3)
N1—C7—C10	118.6 (2)	N5—C21—C24	119.1 (3)
N3—C8—N2	129.1 (3)	N7—C22—N6	128.0 (3)
N3—C8—H8	115.4	N7—C22—H22	116.0
N2—C8—H8	115.4	N6—C22—H22	116.0
N3—C9—C10	121.7 (3)	N7—C23—C28	119.3 (3)
N3—C9—C14	119.1 (3)	N7—C23—C24	121.5 (3)
C10—C9—C14	119.1 (3)	C28—C23—C24	119.2 (3)
C9—C10—C11	119.4 (3)	C23—C24—C25	119.2 (3)
C9—C10—C7	116.1 (2)	C23—C24—C21	115.8 (3)
C11—C10—C7	124.6 (3)	C25—C24—C21	125.0 (3)
C12—C11—C10	120.6 (3)	C26—C25—C24	121.0 (3)
C12—C11—H11	119.7	C26—C25—H25	119.5
C10—C11—H11	119.7	C24—C25—H25	119.5
C11—C12—N4	121.3 (3)	C25—C26—N8	121.1 (3)
C11—C12—C13	119.4 (3)	C25—C26—C27	119.2 (3)
N4—C12—C13	119.2 (3)	N8—C26—C27	119.6 (3)
C14—C13—C12	120.7 (3)	C28—C27—C26	120.7 (3)
C14—C13—H13	119.7	C28—C27—H27	119.7
C12—C13—H13	119.7	C26—C27—H27	119.7
C13—C14—C9	120.8 (3)	C27—C28—C23	120.7 (3)
C13—C14—H14	119.6	C27—C28—H28	119.7
C9—C14—H14	119.6	C23—C28—H28	119.7

### Hydrogen-bond geometry (Å, °)

**Table:**

*D*—H···*A*	*D*—H	H···*A*	*D*···*A*	*D*—H···*A*
N1—H1N···N4^i^	0.93 (4)	2.28 (4)	3.069 (4)	142 (3)
N4—H4A···N3^ii^	0.88	2.33	3.137 (4)	153
N4—H4B···N8^iii^	0.88	2.39	3.178 (4)	149
N8—H8N1···Br1^iv^	0.89 (4)	2.88 (4)	3.739 (4)	163 (3)
N8—H8N2···N7^ii^	0.77 (4)	2.31 (4)	3.053 (4)	162 (4)

Symmetry codes: (i) −*x*+1, −*y*+2, −*z*; (ii) *x*−1, *y*, *z*; (iii) −*x*, −*y*+1, −*z*; (iv) *x*−1, *y*−1, *z*.
